# Evaluation Starch-Based Hemostatic Agents “BioSight” as Adhesion Prevention Barrier Tested in an Adhesion Model in Rats

**DOI:** 10.3390/polym18010033

**Published:** 2025-12-23

**Authors:** Yi-Xin Liu, Chen-Ying Su, Min-Hsuan Yen, Chih-Hwa Chen, Chih-Yu Chen, Hsu-Wei Fang

**Affiliations:** 1 Department of Chemical Engineering and Biotechnology, National Taipei University of Technology, 1, Sec. 3, Zhongxiao E. Rd., Taipei 10608, Taiwan; evieliu@ntut.edu.com (Y.-X.L.); chenying.su@ntut.edu.tw (C.-Y.S.); 2 High-Value Biomaterials Research and Commercialization Center, National Taipei University of Technology, No. 1, Sec. 3, Zhongxiao E. Rd., Taipei 10608, Taiwan; 3 Division of Colorectal Surgery, Department of Surgery, Shuang Ho Hospital, Taipei Medical University, New Taipei City 23561, Taiwan; astrosky0717@gmail.com; 4 Department of Orthopedics, School of Medicine, College of Medicine, Taipei Medical University, Taipei 11031, Taiwan; chihhwache@tmu.edu.tw; 5 Department of Orthopedics, Shuang-Ho Hospital, Taipei Medical University, New Taipei City 23561, Taiwan; 6 School of Biomedical Engineering, College of Biomedical Engineering, Taipei Medical University, Taipei 11031, Taiwan; 7 Institute of Biomedical Engineering and Nanomedicine, National Health Research Institutes, No. 35, Keyan Road, Zhunan Town, Miaoli 35053, Taiwan; 8 Institute of Oral Tissue Engineering and Biomaterials, National Yang Ming Chiao Tung University, Taipei 11221, Taiwan

**Keywords:** BioSight, adhesions, 4DryField^®^ PH, adhesion prevention barrier

## Abstract

**Background:** Postoperative abdominal adhesions are a common and serious complication following abdominal surgery, often leading to chronic pain, bowel obstruction, or infertility. This study aimed to evaluate the efficacy of the new starch-based absorbable hemostatic agent and dressing, BioSight, in comparison with a predicate device (4DryField^®^ PH) for the prevention of abdominal adhesions in a rat model. **Methods:** A total of 90 Sprague–Dawley rats were used to establish an intra-abdominal adhesion model and assigned to the BioSight, 4DryField^®^ PH, or control group. Standardized injuries were created on the cecum and parietal peritoneum, followed by application of the designated materials. Animals were sacrificed at 2, 4, and 12 weeks for macroscopic adhesion scoring and histopathological evaluation. Adhesion area, adhesion strength, and tissue thickness were assessed using established scoring systems, and local healing was examined by H&E staining. All quantitative data were analyzed using one-way ANOVA. **Conclusions:** In a rat peritoneal adhesion model, BioSight exhibited pronounced anti-adhesion efficacy comparable to 4DryField^®^ PH. Macroscopic evaluation showed consistently low adhesion scores (≤0.4) across all time points up to 12 weeks, while histological analysis confirmed reduced adhesion thickness, with BioSight displaying numerically lower values, particularly at early stages (251.3 ± 137.4 µm vs. 323.2 ± 174.6 µm at Week 2). This performance is attributed to rapid in situ hydrogel formation that provides effective temporary tissue separation, limits early fibrin deposition and inflammatory cell infiltration, and supports hemostasis. Importantly, the starch-based hydrogel exhibits a balanced biodegradation profile—persisting long enough to protect injured tissues during the critical inflammatory and fibroproliferative phases, yet undergoing complete enzymatic resorption thereafter without adverse tissue reactions. Collectively, these results highlight the anti-adhesion functionality of BioSight and support the clinical potential of plant-derived starch-based bioresorbable surgical adjuncts.

## 1. Introduction

Postoperative adhesions (POAs) are a common complication, especially Intra-abdominal adhesion is one of the most common and serious postoperative complications following abdominal surgery [1,2,3,4]. It has been reported that approximately 90% of patients develop adhesions of varying severity after abdominal operations, and about 10–20% of them experience clinically significant complications such as chronic abdominal pain, infertility, and intestinal obstruction [3,4]. These postoperative adhesions severely affect patients’ quality of life and impose a considerable healthcare and economic burden. To mitigate adhesion formation, various adhesion barrier products have been developed. These products mainly function by forming a temporary physical barrier at the surgical site to prevent direct contact between injured tissues and to reduce fibrotic responses [4,5]. Commonly used commercial products include absorbable films (e.g., Seprafilm^®^, Interceed^®^), gels or liquid barriers (e.g., Hyalobarrier^®^ Gel), and powder-type barriers (e.g., Arista™ AH, 4DryField^®^ PH, HaemoCer™ Plus) [6,7,8,9,10,11,12].

Each form presents distinct functional characteristics that may influence clinical performance and surgical applicability. Film-type barriers such as Seprafilm^®^ are designed to provide a physical separator between injured tissue surfaces and have demonstrated efficacy in reducing postoperative adhesions in open surgery; however, they can be difficult to position and deploy through narrow laparoscopic ports and on uneven or concave surfaces, limiting their suitability for minimally invasive procedures [13,14]. Additionally, film materials may require careful hydration and fixation to remain in place during the critical early phases of healing.

Gel-type barriers, typically composed of hyaluronic acid and related polymers, offer enhanced conformability to complex anatomical geometries and can be readily injected or spread over large surfaces during laparoscopy. Clinical studies have shown that hyaluronic acid gels reduce adhesion incidence and severity in gynecologic surgery, and their viscoelastic properties help maintain consistent coverage even in deep or irregular cavities [1,15]. However, gel migration from the intended application site and dilution by peritoneal fluids can reduce barrier persistence, potentially compromising effectiveness in certain surgical contexts.

In recent years, powder-type adhesion barriers have attracted increasing interest due to their ease of application, adaptability, and clinical flexibility [4,16]. Unlike film- or gel-type barriers, powders can be directly applied to the surgical field without the need for cutting or fixation, making them particularly suitable for laparoscopic procedures and irregular or complex tissue surfaces [17,18]. Most powder-type products are derived from natural polysaccharides such as starch, chitosan, or oxidized regenerated cellulose [1]. Upon contact with physiological fluids, they rapidly form a hydrogel layer that provides a temporary physical barrier to prevent tissue adhesion while simultaneously exhibiting hemostatic properties. Thus, these materials offer a dual function of hemostasis and anti-adhesion. Certain starch-based powder products such as 4DryField^®^ PH form an in situ gel upon irrigation with saline, combining the ease of powder delivery with the mechanical separation properties of a gel and significantly reducing adhesion formation in clinical settings. While not all powder products confer equivalent anti-adhesive efficacy, formulation-specific performance underscores the importance of product selection based on surgical requirements [19]. In summary, film barriers provide structural separation but are less practical in restricted spaces, gels offer excellent conformability yet may be prone to migration, and powder-type barriers deliver broad surface coverage with simplified laparoscopic application—characteristics that may be particularly advantageous in complex and minimally invasive surgical environments. Furthermore, these materials are generally biocompatible and fully absorbable, degrading completely within few weeks postoperatively without requiring removal or interfering with wound healing [7,12,18,20,21].

Previous studies by Poehnert et al. [11,12] have reported that despite claims made for several commercially available hemostatic powders, 4DryField^®^ PH remains the only starch-based product with verified dual functionality in both hemostasis and adhesion prevention, whereas other hemostatic agents show no significant reduction in peritoneal adhesion formation. 4DryField^®^ PH is a leading plant-based potato starch-based hemostatic and anti-adhesion product, and is also the most commonly used postoperative hemostatic and anti-adhesion product in clinical practice. Numerous preclinical and clinical studies [8,11,12,17,22] have confirmed its reliable hemostatic properties and stable anti-adhesion effects. Therefore, plant starch has emerged as a promising candidate material for preventing postoperative adhesions [21,23,24].

Plant starch powder has good biocompatibility, is fully biodegradable, and has excellent hydrophilicity. Due to its natural source, it does not contain animal proteins or synthetic polymers, thus minimizing the risk of immune responses and infection. Upon contact with water, it rapidly forms a stable hydrogel layer, providing a temporary physical barrier in the early stages of wound healing. This barrier effectively inhibits fibroblast migration and collagen deposition, thereby reducing the formation of fibrous adhesions [5,21,23,24].

BioSight hemostatic powder is an absorbable medical device manufactured in Taiwan, specifically developed for local hemostasis in surgical procedures, and was launched in 2025 (license number MOHW-MDT-MANU-008428). The product states that it is derived from plant starch and is designed to rapidly absorb blood and exudate from bleeding sites, thereby concentrating platelets and clotting factors and promoting thrombus formation. Due to its powder form, BioSight can be easily applied to irregular, wide, or hard-to-reach surfaces, making it suitable for both open and minimally invasive surgeries. After use, the material gradually degrades and is absorbed, requiring no removal. In addition to its known hemostatic function, its physicochemical properties suggest potential applications in surgical fields requiring easy application, surface adhesion, and temporary tissue coverage.

Although BioSight has been developed and used clinically as a hemostatic agent, its physicochemical properties—including rapid fluid absorption, surface adhesion, and temporary coverage of damaged tissue—suggest that it may also act as a physical barrier during the early postoperative healing phase. However, its anti-adhesion potential has not been systematically investigated. With growing interest in powdered materials as flexible and space-saving alternatives to film- or gel-based anti-adhesion barriers, this study focuses on BioSight, also because its composition is similar to 4DryField^®^ PH—both being plant-based starches—but their ability to prevent postoperative adhesions remains to be explored. Therefore, this study will use a rat peritoneal adhesion model [25,26,27,28] to evaluate the anti-adhesion effect of BioSight and compare it with the commercially available reference product, 4DryField^®^ PH. The findings of this study are expected to provide experimental evidence supporting the potential clinical value of plant-derived starch powders as safe, effective, and biocompatible anti-adhesion materials.

## 2. Materials and Methods

### 2.1. Materials

#### 2.1.1. Experimental Animals

According to ISO 10993-6:2016 standards [29], Sprague–Dawley (SD) rats are considered suitable animal models for evaluating the prevention of intra-abdominal adhesions. In this study, female SD rats aged nine weeks were obtained from BioLASCO Taiwan Co., Ltd. (Taipei, Taiwan). All animals were acclimatized under standard laboratory conditions with free access to food and water prior to the surgical procedures. A total of 90 rats were randomly divided into three groups, each receiving a different treatment: the test article (T), the predicate device (P), or the control (C) (sham operation only). Animals were sacrificed at 2, 4, and 12 weeks after surgery for macroscopic and histological evaluations (Table 1).

#### 2.1.2. Test Materials

The following materials were evaluated in this study:

Test article (T): BioSight absorbable hemostatic agent and dressing (Foresight Biomaterial Co., Ltd., Lot No. THA-22102001, Taipei, Taiwan); Predicate device (P): 4DryField^®^ PH Applicator (Lot No. 210505), and Control (C): Sham-operated group without any material application. In addition, the relevant information of the two commercial products used in this study were compared, as shown in Table 2.

### 2.2. Surgical Procedure

Prior to the operation, each rat was anesthetized with Zoletil (1 mg/kg) and xylazine (0.25 mg/kg) via intraperitoneal injection. The abdominal area was shaved and disinfected with povidone–iodine solution, and a midline incision (~5 cm) was made in the lower abdomen. Adhesions were induced by gently abrading a 1 × 2 cm^2^ area on the cecal serosa using sterile dry gauze until petechial bleeding occurred, producing a uniform injury. Additionally, a 1 × 2 cm^2^ defect was created on the parietal peritoneum and inner muscle layer using sharp dissection. The abraded cecum and the injured peritoneum were then sutured together with non-absorbable sutures. Following injury induction, the assigned materials were applied directly to the injured sites, 200 mg per rat, according to group designation. All animals received intramuscular antibiotics once daily for seven days postoperatively. Animals were euthanized at 2, 4, and 12 weeks after surgery for evaluation. The method was based on the research of Poehnert et al. [12].

### 2.3. Clinical Observations and Evaluation Criteria

#### 2.3.1. Clinical Observations

Animals were observed daily throughout the study for general health, clinical symptoms, and potential adverse effects. Any mortality or abnormal findings were documented and subjected to necropsy. Body weights were recorded preoperatively and at 2, 4, and 12 weeks post-surgery. At necropsy, the abdominal cavity was opened, and the extent and strength of adhesions were evaluated using established scoring systems (Table 3 and Table 4).

To reduce detection and assessment bias, animals and specimens were handled using an anonymized coding system. Randomization codes were generated at the time of group assignment and were concealed from personnel performing postoperative evaluations. Macroscopic adhesion scoring was conducted on coded specimens by two independent observers who were blinded to treatment group and who had no involvement in the surgical procedures. When the two observers disagreed, a third blinded observer adjudicated the final score. Photographic documentation of the abdominal cavity was obtained at necropsy to support blinded scoring. Histopathological assessments (including measurement of adhesion tissue thickness and evaluation of inflammatory infiltration) were performed by a board-certified pathologist blinded to group allocation.

#### 2.3.2. Histopathological Evaluation

After sacrifice, the abdominal wall and cecum samples were collected and fixed in 10% neutral buffered formalin (NBF). The tissues were then processed using standard paraffin-embedding procedures, sectioned, and stained with hematoxylin and eosin (H&E). Microscopic examination was performed to assess adhesion tissue thickness and healing status at the injured sites. The histological parameters for wound healing were graded, as summarized in Table 5.

### 2.4. Statistical Analysis

All quantitative data were expressed as mean ± standard deviation (SD). Statistical analyses were conducted using one-way ANOVA followed by Dunnett’s post hoc test (SPSS version 22.0, IBM Corp., Armonk, NY, USA). A p value less than 0.05 was considered statistically significant.

## 3. Results

### 3.1. General Observations and Body Weight Changes

In this study, all animals survived to their scheduled time point and increased in body weight after implantation, as shown in Table 6. No abnormal behaviors and wound infections were found during the test period. These indicated that the animals had undergone a good surgical procedure and good animal care. All animals exhibited normal feeding behavior and mobility, suggesting that neither the test article (BioSight) nor the predicate device (4DryField^®^ PH) induced any systemic or local toxicity.

### 3.2. Macroscopic Adhesion Evaluation

**Figure 1:**
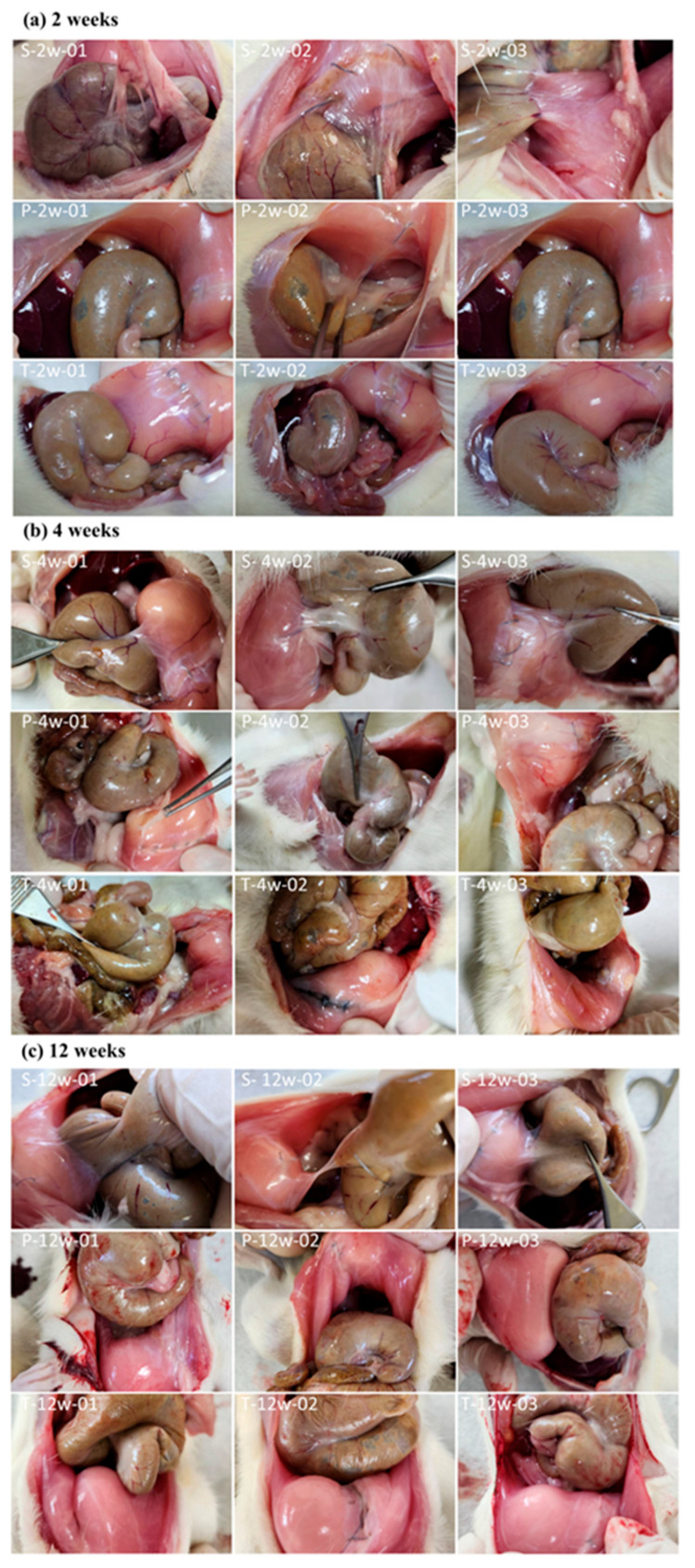
Representative postoperative images of 3 rats out of 10 were selected in (**a**) week 2, (**b**) week 4 and (**c**) week 12. S: Sham Control; P: Predicate Device; T: Test Article.

During necropsy, a midline incision was made in the abdominal wall to evaluate adhesions between the abdominal wall and the cecum. Adhesion area and adhesion strength were assessed using a semi-quantitative scoring system. As shown in Figure 1, 10 animals were included in each group at each time point (weeks 2, 4, and 12), and representative postoperative images from only 3 rats were selected here. In the sham-operated control group, the black suture line in the image represents the abdominal wall wound site. By week 2, significant tissue adhesions were observed between the abdominal wall wound and the cecum (Figure 1a), whereas no significant adhesions were observed when using BioSight or 4DryField^®^ PH. The same situation was observed at week 4 (Figure 1b) and week 12 (Figure 1c).

**Figure 2:**
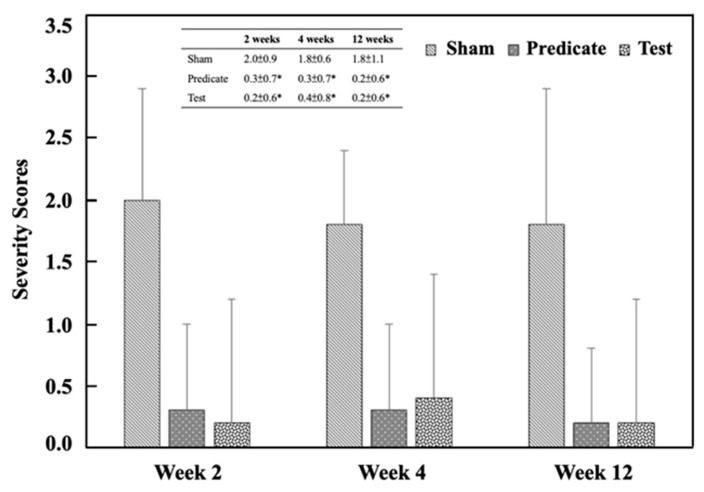
Adhesion area scores at different time points. Significant differences (* *p* < 0.05) compared to the sham group were determined by one-way ANOVA followed by Dunnett’s test.

**Figure 3:**
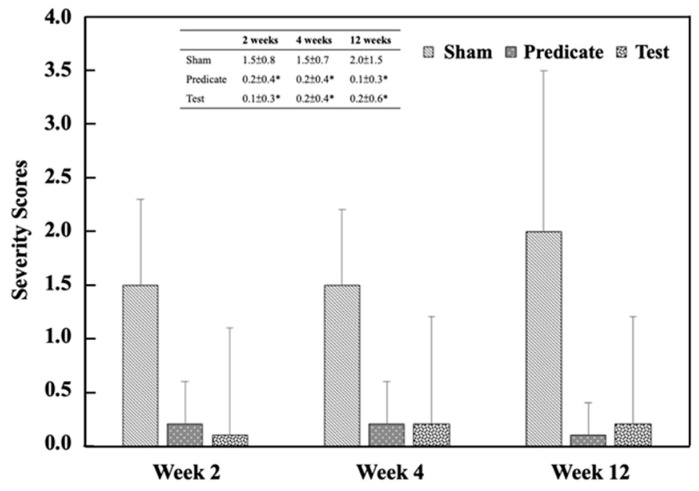
Adhesion strength scores at different time points. Significant differences (* *p* < 0.05) compared to the sham group were determined by one-way ANOVA followed by Dunnett’s test.

Referring to Table 3, the adhesion area was scored in a graded manner, as shown in Figure 2. Both the (T) BioSight and (P) 4DryField^®^ PH groups exhibited significantly lower adhesion area scores than the (S) sham control group at all evaluation time points (*p* < 0.05). The adhesive area score in the sham group was 2.0 ± 0.9, 1.8 ± 0.6 and 1.8 ± 1.1 at weeks 2, 4, and 12, respectively. In contrast, significantly lower scored values were observed in both the test article group (0.2 ± 0.6, 0.4 ± 0.8, 0.3 ± 0.6) and the predicate device group (0.3 ± 0.7, 0.3 ± 0.7, 0.2 ± 0.6) at the same time points. Similarly, the adhesion strength was scored, and the adhesion strength score of the treatment group was also significantly lower than that of the sham-operated control group (Figure 3). The adhesive strength score in the sham group was 1.5 ± 0.8, 1.5 ± 0.7 and 2.0 ± 1.5 at weeks 2, 4, and 12, respectively. In contrast, significantly lower scored values were observed in both the test article group (0.2 ± 0.4, 0.2 ± 0.4, 0.1 ± 0.3) and the predicate device group (0.1 ± 0.3, 0.2 ± 0.4, 0.2 ± 0.6) at the same time points. These findings demonstrate that both materials effectively minimized postoperative adhesion formation and reduced adhesion firmness, consistent with their designed barrier and hemostatic properties.

### 3.3. Histological Evaluation of Adhesive Tissue Thickness

**Figure 4:**
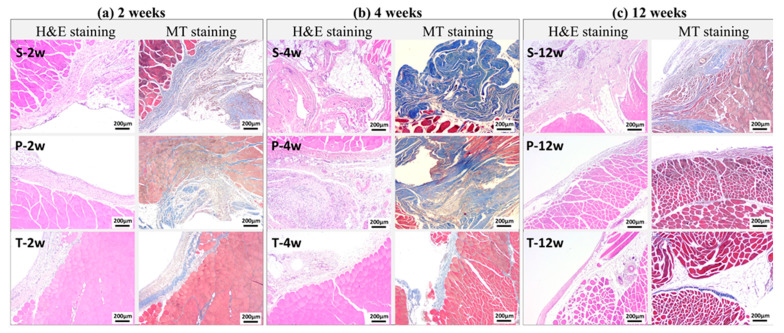
Representative Images of Histopathologic Findings. S: Sham Control; P: Predicate Device; T: Test Article.

**Figure 5:**
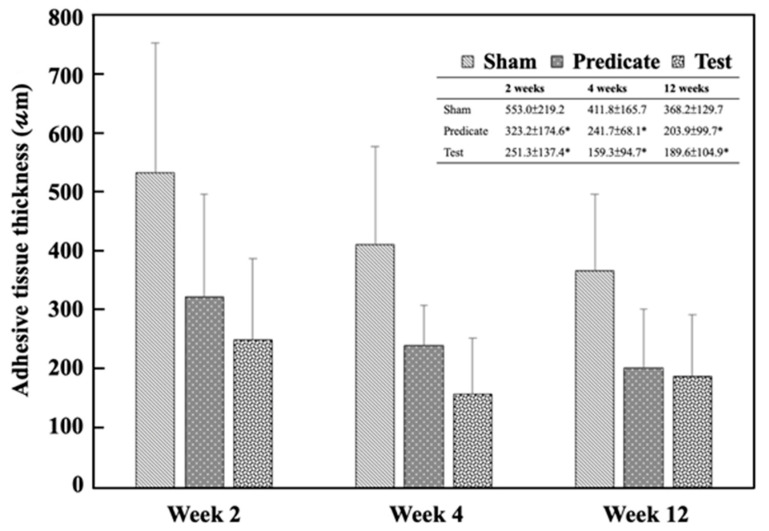
Adhesive tissue thickness in different groups at weeks 2, 4, and 12. Significant differences (* *p* < 0.05) compared to the sham group were analyzed by one-way ANOVA with Dunnett’s test.

Histopathological examination further confirmed the macroscopic findings. Figure 4 presents the corresponding histopathological findings. The adhesive tissue thickness in the sham group was 553.0 ± 219.2 μm, 411.8 ± 165.7 μm, and 368.2 ± 129.7 μm at weeks 2, 4, and 12, respectively. In contrast, significantly lower thickness values were observed in both the test article group (251.3 ± 137.4 μm, 159.3 ± 94.7 μm, 189.6 ± 104.9 μm) and the predicate device group (323.2 ± 174.6 μm, 241.7 ± 68.1 μm, 203.9 ± 99.7 μm) at the same time points (*p* < 0.05) (Figure 5). These data suggest that the BioSight powder effectively inhibited fibroblast infiltration and collagen deposition, thereby reducing adhesion tissue formation and promoting tissue remodeling.

### 3.4. Injury Healing Evaluation

**Figure 6:**
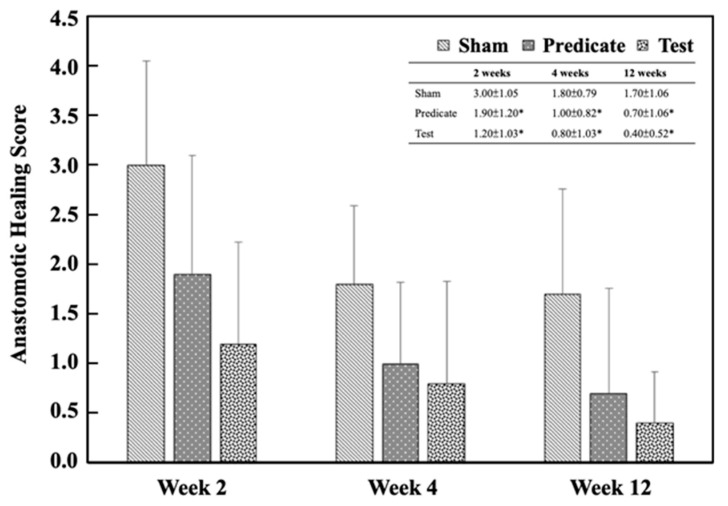
Anastomotic healing scores in the injury sites. Significant differences (* *p* < 0.05) compared to the sham group were analyzed using one-way ANOVA with Dunnett’s test.

Histological assessment of the injured sites was also performed to evaluate tissue regeneration. As shown in Figure 6, both the test article and predicate device groups exhibited significantly improved healing scores compared with the sham group at weeks 2, 4, and 12 (*p* < 0.05), except for the predicate group at week 12, which showed no significant difference.

These results indicate that BioSight not only prevents postoperative adhesions but also supports tissue regeneration and wound healing—likely due to its hydrophilic starch-based composition, which provides a moist microenvironment favorable for tissue repair.

### 3.5. Discussion

Postoperative intraperitoneal adhesions after abdominal surgery remain a major clinical challenge. The results obtained from the rat peritoneal adhesion model (Table 7) demonstrate that both BioSight and 4DryField^®^ PH exhibited pronounced anti-adhesion efficacy, as reflected by reduced adhesion area, adhesion strength, and histological adhesion thickness at all observation time points. These findings confirm that BioSight, originally developed as a hemostatic agent, also provides effective adhesion prevention under in vivo conditions.

The anti-adhesion performance of BioSight can be attributed primarily to its plant-derived starch composition, which rapidly transforms into a hydrogel upon contact with physiological fluids. This in situ-formed gel acts as a temporary physical barrier that separates injured peritoneal surfaces from adjacent tissues during the critical early postoperative phase. By limiting fibrin deposition and inflammatory cell infiltration, the hydrogel effectively disrupts the initial cascade leading to adhesion formation, while simultaneously promoting rapid hemostasis. Although both BioSight and 4DryField^®^ PH are starch-based materials, BioSight demonstrated slightly improved outcomes in adhesion-related parameters, suggesting that formulation-specific properties may influence barrier stability and biological response.

In addition to its barrier function, the starch-based hydrogel exhibits excellent water-retention capacity, creating a moist microenvironment that supports re-epithelialization and overall wound healing. Its high fluid absorption ability concentrates platelets and coagulation factors at the wound site, facilitating rapid hemostasis and early wound stabilization. Importantly, BioSight is gradually biodegraded in vivo by endogenous enzymes such as α-amylase and glucoamylase into non-toxic saccharide byproducts, minimizing the risk of foreign-body reactions and excessive tissue irritation.

A key advantage of the plant-derived starch system lies in its controlled degradation profile, which appears to extend beyond the critical phases of wound healing and fibroproliferation. This degradation timeframe allows the hydrogel barrier to persist long enough to protect traumatized tissues during the period most susceptible to adhesion formation, while still undergoing complete resorption thereafter. In contrast, certain commercially available hydrogel-based anti-adhesion products may degrade or disperse too rapidly in the peritoneal environment, potentially shortening the duration of effective tissue separation and limiting their long-term anti-adhesive efficacy.

Therefore, the hydrogel formed by BioSight not only provides the mechanical separation of injured surfaces but may also modulate the local inflammatory response by restricting cellular infiltration and fibrin accumulation—two key contributors to adhesion development. This dual functionality is consistent with the reduced inflammatory cell presence and improved serosal recovery observed in histological analyses [31,32]. Furthermore, the complete biodegradability and high biocompatibility of starch-based materials ensure safe degradation without interfering with normal tissue repair, supporting their suitability for surgical applications. The combined hemostatic and anti-adhesion effects observed in this study align well with previous reports on polysaccharide-based bioresorbable materials used in adhesion prevention and wound management [33,34].

## 4. Conclusions

The present study demonstrates that BioSight, an absorbable plant-derived starch-based hemostatic agent and dressing, effectively mitigates postoperative intraperitoneal adhesion formation, and may also support physiological wound healing in a rat peritoneal adhesion model. Quantitative macroscopic and histological evaluations revealed that both BioSight and the commercial reference product 4DryField^®^ PH significantly reduced the adhesion area, adhesion strength, and adhesion tissue thickness compared with the sham control group at all evaluated time points.

Beyond its confirmed hemostatic efficacy, BioSight exhibited a clear anti-adhesion effect attributable to its rapid in situ hydrogel formation and sustained barrier function during the critical early postoperative healing phase. Importantly, the controlled biodegradation profile of plant-derived starch allows the material to persist long enough to protect injured peritoneal surfaces throughout the inflammatory and fibroproliferative stages while subsequently undergoing complete enzymatic resorption without eliciting adverse tissue reactions. This degradation behavior addresses a key limitation associated with certain hydrogel-based anti-adhesion products, which may degrade or disperse prematurely in vivo.

Collectively, these findings highlight the dual functional advantages of BioSight—effective hemostasis combined with reliable adhesion prevention—and underscore the importance of material composition and degradation kinetics in anti-adhesion barrier design. The favorable biocompatibility, complete bioresorbability, and ease of application further support BioSight’s potential as a safe and practical anti-adhesion material for abdominal and minimally invasive laparoscopic surgical procedures. This study provides experimental evidence supporting the broader clinical value of plant-derived starch powders as multifunctional bioresorbable surgical adjuncts.

## Figures and Tables

**Table 1:** Treatment groups and sacrifice schedule.

Group	Treatment Material	Sacrifice Time (Weeks)	Number of Animals
(T) Test article	“BioSight” Absorbable hemostatic agent and dressing	2/4/12	10/10/10
(P) Predicate device	4DryField^®^ PH	2/4/12	10/10/10
(C) Sham Control	Sham only	2/4/12	10/10/10
Total			90

**Table 2:** Comparison of the two test agents used in this study.

Parameter	BioSight	4DryField^®^ PH
Manufacturer	Taiwan-based manufacturer(Taipei, Taiwan)	PlantTec Medical GmbH (Lüneburg, Germany)
Product Type	Absorbable hemostatic powder	Absorbable hemostatic and anti-adhesion powder
Primary Intended	Topical hemostasis	Topical hemostasis and adhesion prevention
Material Composition	Plant-derived starch	Plant-derived potato starch
Formulation	Powder	Powder
Mechanism of Hemostasis	Rapid absorption of blood/exudate, concentration of platelets and coagulation factors	Rapid absorption of blood/exudate, concentration of platelets and coagulation factors
In situ Gel Formation	Forms hydrogel upon contact with saline or physiological fluids	Forms hydrogel upon contact with saline or physiological fluids
Anti-adhesion indication	Not indicated; anti-adhesion effect not previously investigated	Indicated and experimentally verified
Evidence for antiadhesion efficacy	Not previously reported	Demonstrated in multiple preclinical and clinical studies
Application suitability	Open and minimally invasive surgery	Open and minimally invasive surgery
Biodegradability	Fully absorbable	Fully absorbable
Removal Required	No	No
Regulatory Status	TFDA-approved medical device (MOHW-MDT-MANU-008428)	CE-marked medical device

**Table 3:** Adhesion area scoring system according to Hoffmann et al. [30].

Severity	Grade	Adhesion Points
Absent	0	No adhesions
Moderate	1	Cecum to bowel adhesion
	2	Cecum to sidewall adhesion over less than 25% of the abraded surface area
	3	Cecum to sidewall adhesion between 25 and 50% of the abraded surface area
Severe	4	Cecum to sidewall adhesion over more than 50% of the surface area

**Table 4:** Adhesion strength scoring system.

Severity	Grade	Type of Adhesion
Absent	0	No adhesions
Moderate	1	Thin, filamentous, easily separated adhesion
	2	Thick adhesions, difficult to dissect, do not tear organ when separated
Severe	3	Thick adhesions not dissectible, tears organ when separated

**Table 5:** Anastomotic healing scoring system.

Parameter	Description	Grading
Inflammation ^1^	Presence of inflammatory cells at the anastomotic site	0 = none; 1 = <10%; 2 = 10–39%; 3 = 40–79%; 4 = 80–100%
Injury recovery ^1^	Evaluation of mucosal and serosal tissue recovery	0 = complete recovery; 1 = no recovery

^1^ The Anastomotic Healing Score was calculated as the sum of both parameters. A lower score indicates better tissue recovery.

**Table 6:** Body weight changes of rats after surgery.

Group	No. of Animals	Body Weight (g)			
		Day 0	Week 2	Week 4	Week 12
(T) BioSight	10	214.2 ± 4.4	244.7 ± 8.5	279.9 ± 15.6	311.5 ± 10.9
(P) 4DryField^®^ PH	10	207.8 ± 6.5	247.1 ± 11.7	275.6 ± 14.1	299.0 ± 12.1
(C) Sham Control	10	218.0 ± 7.1	240.5 ± 13.3	273.9 ± 18.9	304.9 ± 12.5

Values are expressed as mean ± SD (n = 10). Day 0 = before treatment.

**Table 7:** Evaluation of Anti-adhesion Efficacy in a Rat Peritoneal Adhesion Model.

Parameter	BioSight	4DryField^®^ PH
Macroscopic adhesion assessment	Adhesion area and strength were evaluated by adhesion score	
Week 2	0.2 ± 0.6	0.3 ± 0.7
Week 4	0.4 ± 0.8	0.3 ± 0.7
Week 12	0.2 ± 0.6	0.2 ± 0.6
Histological evaluation (H&E staining)	Adhesion thickness evaluated under light microscopy (µm)	
Week 2	251.3 ± 137.4	323.2 ± 174.6
Week 4	159.3 ± 94.7	241.7 ± 668.1
Week 12	189.6 ± 104.9	203.9 ± 99.7

## Data Availability

The original contributions presented in this study are included in the article. Further inquiries can be directed to the corresponding author.
